# Pathology and Classification of SCLC

**DOI:** 10.3390/cancers13040820

**Published:** 2021-02-16

**Authors:** Maria Gabriela Raso, Neus Bota-Rabassedas, Ignacio I. Wistuba

**Affiliations:** Department of Translational Molecular Pathology, The University of Texas MD Anderson Cancer Center, Houston, TX 77030, USA; mbota@mdanderson.org

**Keywords:** pathology and classification of SCLC, biology of SCLC, immune-checkpoint inhibitors in SCLC

## Abstract

**Simple Summary:**

Small cell lung carcinoma (SCLC), is a high-grade neuroendocrine carcinoma defined by its aggressiveness, poor differentiation, and somber prognosis. This review highlights current pathological concepts including classification, immunohistochemistry features, and differential diagnosis. Additionally, we summarize the current knowledge of the immune tumor microenvironment, tumor heterogeneity, and genetic variations of SCLC. Recent comprehensive genomic research has improved our understanding of the diverse biological processes that occur in this tumor type, suggesting that a new era of molecular-driven treatment decisions is finally foreseeable for SCLC patients.

**Abstract:**

Lung cancer is consistently the leading cause of cancer-related death worldwide, and it ranks as the second most frequent type of new cancer cases diagnosed in the United States, both in males and females. One subtype of lung cancer, small cell lung carcinoma (SCLC), is an aggressive, poorly differentiated, and high-grade neuroendocrine carcinoma that accounts for 13% of all lung carcinomas. SCLC is the most frequent neuroendocrine lung tumor, and it is commonly presented as an advanced stage disease in heavy smokers. Due to its clinical presentation, it is typically diagnosed in small biopsies or cytology specimens, with routine immunostaining only. However, immunohistochemistry markers are extremely valuable in demonstrating neuroendocrine features of SCLC and supporting its differential diagnosis. The 2015 WHO classification grouped all pulmonary neuroendocrine carcinomas in one category and maintained the SCLC combined variant that was previously recognized. In this review, we explore multiple aspects of the pathologic features of this entity, as well as clinically relevant immunohistochemistry markers expression and its molecular characteristics. In addition, we will focus on characteristics of the tumor microenvironment, and the latest pathogenesis findings to better understand the new therapeutic options in the current era of personalized therapy.

## 1. Introduction

Lung carcinoma has consistently remained the leading cause of cancer-related death worldwide [1,2]. Currently, in the United States, it ranks as the second most frequent type of new cancer case, both in males and females, accounting for more than 228,800 new cases in 2020 and 12.7% of all new cancer cases [3,4]. Small cell lung carcinoma (SCLC) is a very aggressive, poorly differentiated, and high-grade neuroendocrine carcinoma representing approximately 13% of all lung carcinomas. Most commonly seen in heavy smokers as an advanced stage disease, it clinically presents with early metastatic spread and good responsiveness to initial therapy, and in most patients, it is consistently followed by relapse with a chemo resistant disease [5].

Remarkably, no preneoplastic lesions have been identified in SCLC [6]. This is in stark contrast with the recognition of the diffuse idiopathic pulmonary neuroendocrine cell hyperplasia (DIPNECH) as the precursor lesion for neuroendocrine carcinoids of the lung [7,8], pulmonary neuroendocrine cells (PNECs) are considered putative precursors of small cell lung cancer [9], while the expression of ASCL1 (both in tumors and cell lines) confirms SCLC neuroendocrine lineage [10,11]. However, this concept was challenged with the identification of a distinctive tuft cell variant, showing high POU2F3 expression while lacking neuroendocrine markers, postulating a SCLC-P entity distinctive from the classical neuroendocrine variants, and strongly suggesting a distinct cell-of-origin for this subtype [12]. Alternatively, trans-differentiation towards a tuft cell expression profile from a cell of origin shared with other subtypes remains an alternative explanation. Data from genetically engineered mouse models and human tumors have revealed multiple levels of heterogeneity in SCLC. Additionally, using genetically defined mouse models of SCLC, investigators have uncovered distinct metastatic programs attributable to the cell-of-origin cell-type, concluding that intra-tumoral heterogeneity is influenced by the cell of origin and proposing that SCLC can arise from molecularly distinctive cells. 

## 2. Pathological Classification of SCLC

SCLC was first described in 1879 by Harting and Hesse and classified as a lymphosarcoma [13]; later, in 1926, it was classified as small ‘oat cell’ carcinomas of the lung [14]. From that point forward, it has been historically known as oat cell carcinoma [15,16], and belongs to the neuroendocrine lung tumors group defined in the last WHO Classification of Lung Tumors [17]. In 2015, the World Health Organization (WHO) Classification of Tumors of the Lung, Pleura, Thymus, and Heart was published with numerous important changes from the previous 2004 publication. One of the most significant changes in this edition involves the inclusion of neuroendocrine tumors (NE) grouped together in one category, recognizing four major NE tumors: typical carcinoid tumor (TC), atypical carcinoid tumor (AC), SCLC, and large cell NE carcinoma (LCNEC) [17]. The current sub-classification recognizes two subtypes: pure SCLC and combined SCLC, the latter is determined by the presence of features of a different histological carcinoma, non-small cell lung carcinoma (NSCLC), variant or at least containing 10% of large cell carcinoma component. Up to 28% of surgically resected SCLC tumors are combined SCLC, and of these, 16% belong to the large cell carcinoma variant [18,19]. 

## 3. Macroscopic Features of SCLC

As a highly aggressive disease, SCLC is found disseminated in the initial clinical presentation in the majority of cases, and surgery is limited to a small subgroup of patients with confined disease [20]. Usually, these tumors undergo surgical resection only when diagnosis of SCLC has not been previously established, and it is from those infrequent surgical specimens that a macroscopic description of SCLC tumors is described. These tumors are grossly identifiable as fleshy, ranging from a white and grayish to a light tan color, soft and friable irregular masses, with necrotic cut surface areas. The majority of them are situated in hilar or perihilar areas, with less than 5% of the cases presenting in peripheral locations. Invasion into the peribronchial tissue and lymph node is often grossly identifiable, typically spreading in circumferentially along the submucosa of the bronchi.

## 4. Microscopic Features of SCLC

### 4.1. Cytological Features

**Figure 1:**
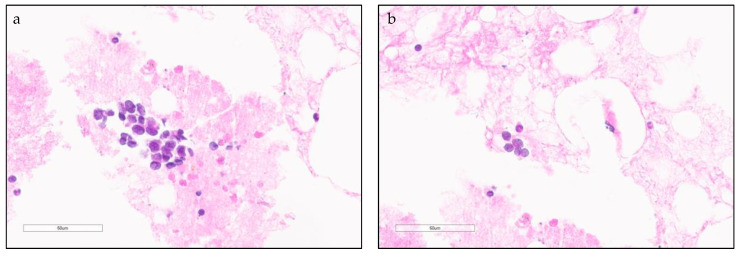
Small cell lung carcinoma (SCLC) cytological and histological characteristics. (**a**,**b**) High power field view of SCLC in cellular aspirates. Loosely arranged small blue cells with scant cytoplasm and fine chromatin features (arrow).

Cytology is a very reliable method to establish a SCLC diagnosis and often a valuable complement to bronchial biopsies where crush artifacts may hamper a definitive diagnosis. The most common cytologic feature is highly cellular aspirates with presence of small blue cells with very scant or null cytoplasm, loosely arranged or in a syncytial pattern (Figure 1). Within tightly cohesive sheets, nuclear molding is well developed. Additionally, a background of single cells, doublets, triplets, and small cell cords with extensive necrosis are characteristic of this entity. The mitotic rate is usually very high (10 mitosis per 10 high power fields) and chromatin smearing is very frequent. Well-preserved cells show the characteristic “salt and pepper” chromatin patter, while less well-preserved cells show dark blue chromatin. Cytoplasmic globules, also named paranuclear blue bodies, are considered distinctive of a SCLC diagnosis [21,22].

### 4.2. Histopathological Features

**Figure 2:**
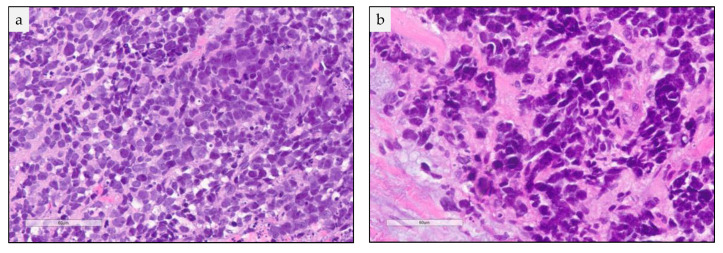
Small cell lung carcinoma (SCLC) cytological and histological characteristics. High power field view of a formalin fixed paraffin embedded tissue H&E stained slide showing in (**a**). sheets of small cells with scant cytoplasm and nuclear molding and (**b**). Sheets of tightly packed small cells and intratumoral necrosis.

**Figure 3:**
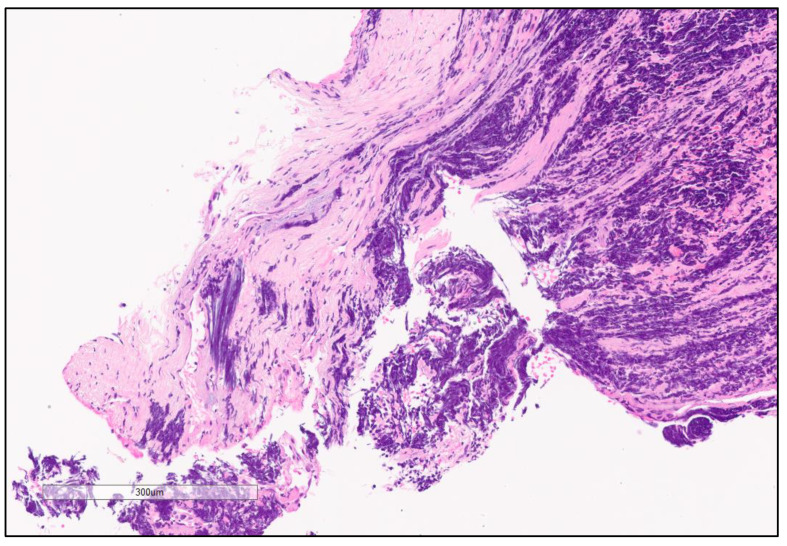
Chromatin smearing “crush artifact”. High magnification showing Small cell lung carcinoma (SCLC) with extensive chromatin smearing “crush artifact”.

**Figure 4:**
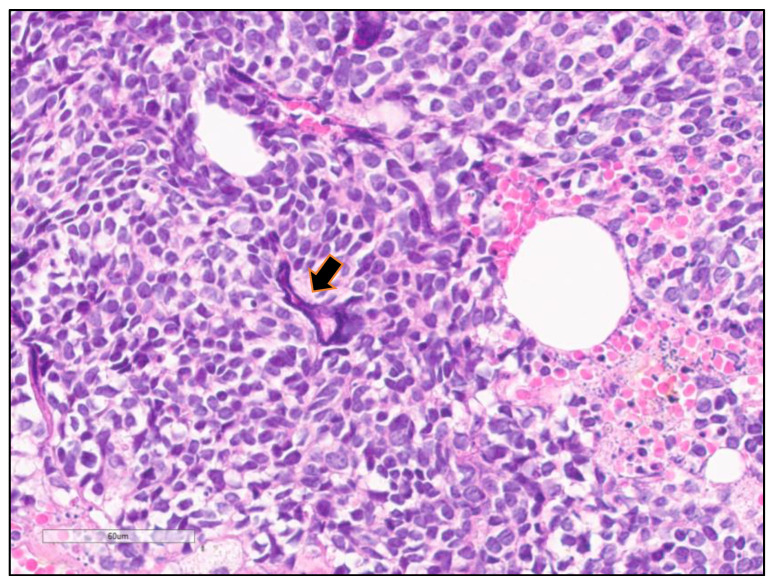
Azzopardi phenomenon. High magnification showing Small cell lung carcinoma (SCLC) with basophilic nuclear debris encrustation of the blood vessel wall (arrow).

Hematoxylin and eosin (H&E) assessment of a SCLC tumor under a light microscope main features is defined by the presence of diffuse sheets of small round to fusiform cells, with scant cytoplasm, and inconspicuous or absent nucleoli with finely granular nuclear chromatin (Figure 2). Nuclear chromatin smearing, also called crushed artifact, is a common feature together with nuclear molding (Figure 2 and Figure 3). Extensive intratumoral necrosis is often recognized, as well as the high mitotic index that defines the tumor (average of 40 mitosis/mm^2^ area) [18,23,24] (Figure 2). In addition to the common diffuse sheet-like growth pattern, SCLC tumor can demonstrate other growth features as peripheral palisading, streams, ribbons, organoid nesting, and rosettes [18,23]. Another characteristic feature is the frequent basophilic nuclear debris encrustation of the blood vessel wall, widely known as Azzopardi effect (Figure 4) [25,26]. Larger specimens may present a variety of cellular characteristics like larger cells, pleomorphic cells, giant tumor cells, and dispersed chromatin with prominent nucleoli, among others. 

SCLC can be observed as a pure SCLC histology or combined with NSCLC histology features. The latter refers to the admixture of NSCLC elements; where adenocarcinoma, squamous cell carcinoma (SCC), and large cell carcinoma can be seen, as well as, although at a lower frequency, giant cell carcinoma and spindle cell carcinoma [17]. To establish a diagnosis of combined SCLC and large cell carcinoma, large cells have to constitute at least 10% of the tumor; however, no assessment of the percentage of the secondary histology is necessary to render a combined SCLC diagnosis for other NSCLC histologies [19]. 

### 4.3. Immunohistochemistry

The histopathological diagnosis is based on light microscopy using routine H&E stained slides, as stated in the current 2015 WHO classification. Immunohistochemistry (IHC) assays are required when confirmation of equivocal features is necessary or when a differential diagnosis must be addressed, especially in small biopsies with crush artifacts (Table 1).

SCLC stains positively for low molecular weight keratins, showing a dot-like paranuclear or diffuse cytoplasmic staining, and is negative for high molecular weight keratins. SCLC shows a variable expression of neuroendocrine differentiation markers such as neural cell adhesion molecule (NCAM/CD56), chromogranin, synaptophysin, and insulinoma-associated protein 1 (INSM1), and usually presents lower protein levels than low-to-intermediate grade neuroendocrine tumors. It is important to note that a minority of SCLCs are negative for all standard neuroendocrine markers. Importantly, immunohistochemistry has only a limited role in distinguishing LCNEC and SCLC. One potentially helpful, although with low-sensitivity, feature is focal and weak napsin A immunoreactivity, which can be seen in up to 15% of LCNEC [27] while SCLC is consistently negative for this marker. Thus, when present, weak napsin A labeling in a high-grade NE carcinoma may favor the diagnosis towards LCNEC. Thyroid transcription factor-1 (TTF-1) is a homeodomain-containing transcription factor selectively expressed in pulmonary adenocarcinomas, thyroid tumors, and small cell carcinomas. Interestingly, TTF-1 is expressed in close to 90% of SCLC, which can be useful for differentiating SCLC from neuroendocrine cancers not originating from the lung.

High Ki67 (MIB-1) labeling index (>50%, usually 70–100%) is a hallmark of SCLC, helping in distinguishing it from low- and intermediate-grade neuroendocrine cancers [28]. Other markers used to characterize SCLC are: B-cell lymphoma 2 (BCL2; expressed often), P16 nuclear staining (expressed in 95–100% of cases), tyrosine-protein kinase KIT (CD117/c-KIT; expressed in about 60% of cases, retinoblastoma protein (P-RB; always negative), and homeobox protein orthopedia (OTP, always negative) [29].

## 5. Differential Diagnosis of SCLC

Although SCLC has a very well-defined diagnostic criteria, sampling issues, fixation artifacts, and the morphologic variability of pure SCLC tumor cells, can all potentially make the diagnosis of SCLC challenging.

The current WHO classification of lung cancer recognizes four neuroendocrine tumors, namely typical carcinoid tumor (TC), atypical carcinoid tumor (AC), SCLC, and large cell NE carcinoma (LCNEC). Neuroendocrine tumors have a wide differential diagnosis based on their mitotic rate and necrosis extension. Other histologies, such as basaloid squamous cell carcinoma, small round cell sarcoma, metastatic breast carcinoma, and non-Hodgkin’s lymphoma, may be considered in the differential diagnosis of SCLC. In these cases, it is important to perform IHC in order to confirm its neuroendocrine differentiation and epithelial nature, detect other differentiation markers, as well as to assess the site of origin and proliferation rate. As an example, p63 and TTF-1 expression is useful in distinguishing SCLC from poorly differentiated nonkeratinizing SCC: p63 positive and TTF-1 negative expression consequently indicates a poorly differentiated nonkeratinizing SCC, while an opposite immunostaining pattern identifies a SCLC diagnosis.

## 6. Landscape of the Immune Tumor Microenvironment in SCLC

Current evidence shows that the immune system is capable of generating antitumor responses against various tumors, including lung cancer, suggesting that immunotherapy may be a viable therapeutic approach for patients with SCLC [30]. Increasing evidence shows that the immune system is involved in the pathophysiology of SCLC [31]. Moreover, confirmation that SCLC is immunogenic comes from the relationship between immune activity and prognosis. For instance, more infiltrating CD45+ T-cells in SCLC tumors were found to be a predictor of better OS, independently of stage and performance status. In addition, more effector T-cells were found in limited stage disease (LD) SCLC compared with extended stage disease (ED) SCLC cases, and higher effector-to-regulatory T-cell ratios were associated with longer survival [22,30,32]. 

As it is known, NSCLC patients with a high mutation burden have been shown to be particularly sensitive to immunotherapeutic agents that inhibit the PD-1 pathway [30,33,34], indicating that SCLC could potentially benefit from immunotherapy treatments, given its high mutation burden [35]. However, several mechanisms of immune resistance have been identified in SCLC, such as downregulation of major histocompatibility complex (MHC) antigens I and II, low PD-L1 expression, increase in regulatory T-cells, and poor tumor infiltration of effector T-cells, partially due to a lack of vasculature surrounding the tumor [36].These features likely diminish the response in SCLC compared to the observed immunotherapeutic response in NSCLC. 

Immunotherapies targeted against programmed death ligand 1 (PD-L1) and its receptor (PD-1) have improved survival in a subset of NSCLC patients. PD-L1 protein expression has emerged as a biomarker that predicts which patients are more likely to respond to immunotherapy. Noticeably, PD-L1 expression has been demonstrated in SCLC, although at lower levels than NSCLC. In one retrospective study of 102 SCLC specimens, PD-L1 expression was observed in 72% of tumor cells and was associated with significantly longer overall survival (OS) [37]. In contrast, another study involving 94 clinical cases of small cell NE carcinoma found PD-L1 expression in 19% of cases, and only expressed in stromal cells [38]. Similarly, others have reported PD-L1 expression in tumor cells, ranging from 27% to 89% [39,40]. The inconsistency of these findings is most likely related to differences in PD-L1 detection methods, the utilization of different IHC platforms and antibodies, as well as the lack of standardized scoring methods [41].

Regulatory CD4^+^ T-cells play a major role suppressing other immune cells, lessening the immune response. A study demonstrated the ability of SCLC cell lines to induce a de novo differentiation from activated CD4^+^ T-cells to regulatory CD4^+^ T-cells (FOXP3^+^ T-cells), by secretion of IL-15 [42]. The same study, including data from a cohort of 65 SCLC patients, demonstrated the presence of IL-15 in SCLC biopsies, and a positive correlation between increase in tumor infiltration of FOXP3 T-cells and worse OS [42], opening new avenues for future immune therapies against SCLC.

MHC proteins are usually present at the cell surface of tumor cells, playing a central role in antigen presentation to activate cytotoxic T-cells and elicit antitumor immune response. Strikingly, it is another component of SCLC suppressed immunogenicity, as both MHC class I and II expression have been reported to be decreased in SCLC [43,44]. 

Overall, there is solid evidence that SCLC has the potential to be immunogenic. This could be achieved by counteracting the immunosuppressive nature of this tumor type, and for this purpose, further research is needed to understand the specific pathways that drive SCLC-mediated immunosuppression. 

## 7. SCLC Tumor Heterogeneity 

Evidence of intra-tumoral heterogeneity in SCLC was demonstrated many years ago by the diverse expression of antigens detectable with monoclonal antibodies [45]. Contemporary work from animal models such as genetically engineered mouse models (GEMMs) and patient-derived xenograft/cell-derived xenograft (PDX/CDX) models has shown new levels of cellular complexity in SCLC. The presence of several subpopulations of SCLC cells and their functional interactions may explain the outstanding plasticity of SCLC tumors and their striking metastatic potential [46,47]. Additionally, SCLC tumors that look similar at the histopathological level may represent distinct subtypes of tumors, and these differences have an impact on the response to specific therapeutic agents. A better understanding of genetic and cellular heterogeneity will guide the development of personalized approaches to help SCLC patients [46].

## 8. Genetic Variation of SCLC

SCLC is a molecularly complex disease comprising numerous genetic alterations, including variations in tumor suppressor genes, copy number variation, somatic mutations in transcription factors, chromatin modification, and receptor tyrosine kinases or their downstream signaling components [48] (Table 2). Notably, 95% of tumors reveal loss of *Retinoblastoma 1* (*RB1*) and more than 65% present *TP53* mutations [49,50,51,52,53]. In addition, frequent allelic loss involving chromosome arm 3p deletion has been reported in 91% of SLCC tumors [54]. Mutations in *v-myc avian myelocytomatosis viral oncogene homolog* (*MYC*) family genes, *BCL2*, *Phosphatase and tensin homolog* (*PTEN*), *Slit homolog 2* (*SLIT2*), *CREB binding protein* (*CREBBP*), *Ephrin type-A receptor 7* (*EPHA7*), and *Fibroblast Growth Factor Receptor 1* (*FGFR1*) mutations have been identified [35]. Other molecular abnormalities, such as increased expression of c-KIT, amplification of *MYC* family members (*MYC*, *MYC lung carcinoma-derived homolog 1* [*MYCL1*], *MYC neuroblastoma-derived homolog* [*MYCN*]), and loss of *PTEN* have also been described in subsets of SCLC [54,55,56,57,58]. 

Recently, comprehensive genomic research studies with cell lines, GEMMs, and PDX models have revealed biologically heterogenous SCLC subtypes (Table 3), based on mRNA expression profiles defined by the differential expression of four key transcription regulators: *achaete-scute homologue 1* (*ASCL1*; also known as *ASH1*), *neurogenic differentiation factor 1* (*NeuroD1*), *yes-associated protein 1* (*YAP1*), and *POU class 2 homeobox 3* (*POU2F3*). As a result of such studies, recent consensus suggested grouping SCLC into four subtypes defined by mRNA expression of *ASCL1*, *NEUROD1*, *POU2F3*, and *YAP1*. Consequently, a working nomenclature for SCLC subtypes defined by the relative expression of these four factors was proposed, classifying cases as: ASCL1 high (SCLC-A), NEUROD1 high (SCLC-N), POU2F3 high (SCLC-P), and YAP1 high (SCLC-Y) [12,59,60,61]. SCLC-Y is characterized by WT *RB1* enrichment [62] and low or absent expression of *ASCL1*, *NEUROD1*, and other neuroendocrine markers, accounting for approximately 5% to 10% of SCLC tumors. In most instances of combined SCLC with NSCLC histologies, there is a strong evidence of clonality between SCLC and NSCLC components, likely indicating a common precursor cell in such cases [12,63]. When comparing the morphological features and considering the similar genetics and genomics characteristics between SCLC-Y (WT *RB1*) and LCNEC (WT *RB1*, WT *KEAP1*, WT *STK11*) subtypes, together with a low or absent expression of *ASCL1* and *NEUROD1*, it has been suggested that these two lung cancer subtypes belong to a single entity [62]; thus, implying potential impact in molecular classification and clinical implications. Recently, a first comprehensive IHC-based study in patient samples has confirmed highly distinct characteristics of SCLC tumors expressing *ASCL1* (SCLC-A) and/or *NEUROD1* (SCLC-N) (86%) compared to SCLC lacking these markers (14%) [59]. The former is associated with a high NE program (NE-markers high/TTF-1 high/DLL3 high) and a pure SCLC histology, whereas the latter exhibits an enrichment in a combined SCLC histology. The same study also confirmed a highly distinctive nature for *POU2F3*-expressing tumors (SCLC-P), which account for 7% of SCLC [59]. Using tumor expression data and non-negative matrix factorization, Gay et al. identified four SCLC subtypes defined largely by differential expression of transcription factors ASCL1, NEUROD1, and POU2F3 or low expression of all three transcription factor signatures accompanied by an Inflamed gene signature (SCLC-A, N, P, and I, respectively). Stating that the SCLC-I subgroup may benefit from the addition of immunotherapy to chemotherapy, while the other subtypes each have distinct vulnerabilities, including to inhibitors of PARP, Aurora kinases, or BCL-2 [64]. 

Albeit uncommon, transformation of NSCLC into SCLC has been observed as a resistance mechanism upon treatment of *EGFR*-mutated NSCLC with *EGFR* tyrosine kinase inhibitors (TKI) (3% to 10% of *EGFR*-TKI resistant cases) [65,66]. Interestingly, NSCLC to SCLC transformation is dependent on EGFR mutation, as only rare instances of such transformation have been reported in EGFR-WT NSCLC, and it is often accompanied by mutations in *TP53*, *Rb1*, and *PIK3CA* [66,67,68,69,70]. 

## 9. Conclusions

SCLC represents one of the most lethal forms of cancer, with limited successful therapeutic options and consequently presenting striking low survival rates in late stages. It is reckoned as a high-grade neuroendocrine lung carcinoma and it is classified as either pure SCLC or combined SCLC, the latter when a portion of the tumor shows NSCLC features. Although the histological diagnosis is based mostly in H&E specimens, IHC can prove very helpful in the differential diagnosis setting. Intratumoral heterogeneity is thought to be related with its remarkable plasticity and striking metastatic potential. Histologically similar SCLC tumors may actually represent distinct subtypes of tumors, linked to disparate response outcomes to specific therapeutic agents. Recent molecular classifications approaches are paving the way to a deeper understanding of this entity and to related potential treatment approaches. Further knowledge of this entity, including both its genetic features and cellular heterogeneity, as well as its tumor microenvironment, will guide the development of personalized therapeutic strategies for SCLC patients. In the authors opinion, an exciting new era of molecular driven treatment decisions is finally foreseeable in the near future for SCLC patients. 

## Figures and Tables

**Table 1:** Immunohistochemistry (IHC) markers and immunohistochemical characteristics of SCLC.

Marker	Stain	Comments
Keratin (MW^low^)	+	Paranuclear/Cytoplasmatic diffuse
Keratin (MW^high^)	-	Not expressed in SCLC
Ki67	+	At high levels, hallmark of SCLC vs NE tumors
NCAM/CD56	+	Variable/Lower compared to other NE tumors
Chromogranin	+	Variable/Lower compared to other NE tumors
Synaptophysin	+	Variable/Lower compared to other NE tumors
INSM1	+	Variable/Lower compared to other NE tumors
Napsin A	-	Presence of this marker favors LCNEC diagnosis
TTF-1	+	Useful SCLC diagnosis vs NE tumors not originated in the lung
BCL-2	+	Often expressed in SCLC
P16	+	Expressed in >95% of SCLC
CD117/KIT	+	Expressed in 60% of SCLC
P-RB	-	Never expressed in SCLC
OTP	-	Never expressed in SCLC

**Table 2:** Genomic alterations and markers for Small cell lung carcinoma (SCLC).

Marker	Genetic Alteration	Comments
RB1	Loss	95% of SCLC
TP53	Mutation	65% of SCLC
Chromosome arm 3p	Deletion	91% of SCLC
MYC	Mutation	Variable
BCL2	Mutation	Variable
PTEN	Mutation	Variable
SLIT2	Mutation	Variable
CREBBP	Mutation	Variable
EPHA7	Mutation	Variable
FGFR1	Mutation	Variable
c-KIT	Increased Expression	Described in some SCLC subsets
MYC, MYCL1, MYCN	Amplification	Described in some SCLC subsets
PTEN	Loss	Described in some SCLC subsets

**Table 3:** Molecular classification of Small cell lung carcinoma (SCLC) subsets.

SCLC Subtype	Markers	Characteristics
SCLC-A	ASCL1	Pure SCLC histology; NE-markers^high/^TTF-1^high/^DLL3^high^
SCLC-N	NEUROD1	Enrichment in combined SCLC histology; NE-markers^high/^TTF-1^high^/DLL3^high^
SCLC-P	POU2F3	Low or absent expression of ASCL1 and NEUROD1, NE-markers^low^/TTF-1^low^/DLL3^low^
SCLC-Y	YAP1	Low or absent expression of ASCL1 and NEUROD1; RB1*^wt^*
SCLC-I	Inflamed Gene signature	Low expression of ASCL1, NEUROD1, and POU2F3

## References

[1] Bray F., Ferlay J., Soerjomataram I., Siegel R.L., Torre L.A., Jemal A. (2018). Global cancer statistics 2018: GLOBOCAN estimates of incidence and mortality worldwide for 36 cancers in 185 countries. CA Cancer J.Clin..

[2] International Agency for Research on Cancer (WHO). https://gco.iarc.fr/today/home.

[3] American Cancer Society Key Statistics for Small Cell Lung Cancer. https://www.cancer.org/cancer/lung-cancer/about/key-statistics.html.

[4] National Health Institute Surveillance, Edipemiology, and End Results Program (SEER). https://seer.cancer.gov/statfacts/html/lungb.html.

[5] Gibbons D.L., Byers L.A., Kurie J.M. (2014). Smoking, p53 mutation, and lung cancer. Mol. Cancer Res..

[6] Kadara H., Scheet P., Wistuba I.I., Spira A.E. (2016). Early events in the molecular pathogenesis of lung cancer. Cancer Prev Res..

[7] Carr L.L., Chung J.H., Duarte Achcar R., Lesic Z., Rho J.Y., Yagihashi K., Tate R.M., Swigris J.J., Kern J.A. (2015). The clinical course of diffuse idiopathic pulmonary neuroendocrine cell hyperplasia. Chest.

[8] Rossi G., Cavazza A., Spagnolo P., Sverzellati N., Longo L., Jukna A., Montanari G., Carbonelli C., Vincenzi G., Bogina G. (2016). Diffuse idiopathic pulmonary neuroendocrine cell hyperplasia syndrome. Eur. Respir. J..

[9] Chen H.J., Poran A., Unni A.M., Huang S.X., Elemento O., Snoeck H.W., Varmus H. (2019). Generation of pulmonary neuroendocrine cells and SCLC-like tumors from human embryonic stem cells. J. Exp. Med..

[10] Augustyn A., Borromeo M., Wang T., Fujimoto J., Shao C., Dospoy P.D., Lee V., Tan C., Sullivan J.P., Larsen J.E. (2014). ASCL1 is a lineage oncogene providing therapeutic targets for high-grade neuroendocrine lung cancers. Proc. Natl. Acad. Sci. USA.

[11] Borromeo M.D., Savage T.K., Kollipara R.K., He M., Augustyn A., Osborne J.K., Girard L., Minna J.D., Gazdar A.F., Cobb M.H. (2016). ASCL1 and NEUROD1 Reveal heterogeneity in pulmonary neuroendocrine tumors and regulate distinct genetic programs. Cell Rep..

[12] Huang Y.H., Klingbeil O., He X.Y., Wu X.S., Arun G., Lu B., Somerville T.D.D., Milazzo J.P., Wilkinson J.E., Demerdash O.E. (2018). POU2F3 is a master regulator of a tuft cell-like variant of small cell lung cancer. Genes Dev..

[13] Härting F.H., Hesse W., Gedruckt bei L. (1879). Der Lungenkrebs, die Bergkrankheit in den Schneeberger Gruben.

[14] Bernard W.G. (1926). The nature of the ’oat-celled sarcoma’ of the mediastinum. J. Pathol. Bacteriol..

[15] Azzopardi J.G. (1959). Oat-cell carcinoma of the bronchus. J. Pathol. Bacteriol..

[16] Watson W.L., Berg J.W. (1962). Oat cell lung cancer. Cancer.

[17] Travis W.D., Brambilla E., Nicholson A.G., Yatabe Y., Austin J.H.M., Beasley M.B., Chirieac L.R., Dacic S., Duhig E., Flieder D.B. (2015). The 2015 world health organization classification of lung tumors: Impact of genetic, clinical and radiologic advances since the 2004 classification. J. Thorac. Oncol..

[18] Nicholson S.A., Beasley M.B., Brambilla E., Hasleton P.S., Colby T.V., Sheppard M.N., Falk R., Travis W.D. (2002). Small cell lung carcinoma (SCLC): A clinicopathologic study of 100 cases with surgical specimens. Am. J. Surg. Pathol..

[19] Travis W.D. (2012). Update on small cell carcinoma and its differentiation from squamous cell carcinoma and other non-small cell carcinomas. Mod. Pathol..

[20] Hamilton G., Rath B. (2017). Mesenchymal-epithelial transition and circulating tumor cells in small cell lung cancer. Adv. Exp. Med. Biol..

[21] Mullins R.K., Thompson S.K., Coogan P.S., Shurbaji M.S. (1994). Paranuclear blue inclusions: An aid in the cytopathologic diagnosis of primary and metastatic pulmonary small-cell carcinoma. Diagn. Cytopathol..

[22] Wang W., Hodkinson P., McLaren F., Mackean M.J., Williams L., Howie S.E.M., Wallace W.A.H., Sethi T. (2013). Histologic assessment of tumor-associated CD45^+^ cell numbers is an independent predictor of prognosis in small cell lung cancer. Chest.

[23] Travis W.D. (2009). Lung tumours with neuroendocrine differentiation. Eur. J. Cancer.

[24] Travis W.D. (2011). Pathology of lung cancer. Clin. Chest Med..

[25] Moran C.A., Suster S., Coppola D., Wick M.R. (2009). Neuroendocrine carcinomas of the lung: A critical analysis. Am. J. Clin. Pathol..

[26] Weissferdt A. (2020). Neuroendocrine tumors of the lung. Diagnostic Thoracic Pathology.

[27] Baine M.K., Sinard J.H., Cai G., Homer R.J. (2020). A semiquantitative scoring system may allow biopsy diagnosis of pulmonary large cell neuroendocrine carcinoma. Am. J. Clin. Pathol.

[28] Zheng M. (2016). Classification and pathology of lung cancer. Surg. Oncol. Clin. N. Am..

[29] Thunnissen E., Borczuk A.C., Flieder D.B., Witte B., Beasley M.B., Chung J.H., Dacic S., Lantuejoul S., Russell P.A., den Bakker M. (2017). The use of immunohistochemistry improves the diagnosis of small cell lung cancer and its differential diagnosis. An international reproducibility study in a demanding set of cases. J. Thorac. Oncol..

[30] Horn L., Reck M., Spigel D.R. (2016). The future of immunotherapy in the treatment of small cell lung cancer. Oncologist.

[31] Tani T., Tanaka K., Idezuka J., Nishizawa M. (2008). Regulatory T cells in paraneoplastic neurological syndromes. J. Neuroimmunol..

[32] Koyama K., Kagamu H., Miura S., Hiura T., Miyabayashi T., Itoh R., Kuriyama H., Tanaka H., Tanaka J., Yoshizawa H. (2008). Reciprocal CD4^+^ T-cell balance of effector CD62L^low^ CD4^+^ and CD62L^high^CD25^+^ CD4^+^ regulatory T cells in small cell lung cancer reflects disease stage. Clin. Cancer Res..

[33] Rizvi N.A., Hellmann M.D., Snyder A., Kvistborg P., Makarov V., Havel J.J., Lee W., Yuan J., Wong P., Ho T.S. (2015). Mutational landscape determines sensitivity to PD-1 blockade in non–small cell lung cancer. Science.

[34] Willis C., Fiander M., Tran D., Korytowsky B., Thomas J.-M., Calderon F., Zyczynski T.M., Brixner D., Stenehjem D.D. (2019). Tumor mutational burden in lung cancer: A systematic literature review. Oncotarget.

[35] Peifer M., Fernández-Cuesta L., Sos M.L., George J., Seidel D., Kasper L.H., Plenker D., Leenders F., Sun R., Zander T. (2012). Integrative genome analyses identify key somatic driver mutations of small-cell lung cancer. Nat. Genet..

[36] Hamilton G., Rath B. (2019). Immunotherapy for small cell lung cancer: Mechanisms of resistance. Expert Opin. Biol. Ther..

[37] Ishii H., Azuma K., Kawahara A., Yamada K., Imamura Y., Tokito T., Kinoshita T., Kage M., Hoshino T. (2015). Significance of programmed cell death-ligand 1 expression and its association with survival in patients with small cell lung cancer. J. Thorac. Oncol..

[38] Schultheis A.M., Scheel A.H., Ozretić L., George J., Thomas R.K., Hagemann T., Zander T., Wolf J., Buettner R. (2015). PD-L1 expression in small cell neuroendocrine carcinomas. Eur. J. Cancer.

[39] Komiya T., Madan R. (2015). PD-L1 expression in small cell lung cancer. Eur. J. Cancer.

[40] Ott P.A., Fernandez M.E.E., Hiret S., Kim D.-W., Moss R.A., Winser T., Yuan S., Cheng J.D., Piperdi B., Mehnert J.M. (2015). Pembrolizumab (MK-3475) in patients (pts) with extensive-stage small cell lung cancer (SCLC): Preliminary safety and efficacy results from KEYNOTE-028. J. Clin. Oncol..

[41] Yu H., Boyle T.A., Zhou C., Rimm D.L., Hirsch F.R. (2016). PD-L1 expression in lung cancer. J. Thorac. Oncol..

[42] Wang W., Hodkinson P., McLaren F., MacKinnon A., Wallace W., Howie S., Sethi T. (2012). Small cell lung cancer tumour cells induce regulatory T lymphocytes, and patient survival correlates negatively with FOXP3^+^ cells in tumour infiltrate. Int. J. Cancer.

[43] Doyle A., Martin W.J., Funa K., Gazdar A., Carney D., Martin S.E., Linnoila I., Cuttitta F., Mulshine J., Bunn P. (1985). Markedly decreased expression of class I histocompatibility antigens, protein, and mRNA in human small-cell lung cancer. J. Exp. Med..

[44] He Y., Rozeboom L., Rivard C.J., Ellison K., Dziadziuszko R., Yu H., Zhou C., Hirsch F.R. (2017). MHC class II expression in lung cancer. Lung Cancer.

[45] Fargion S., Carney D., Mulshine J., Rosen S., Bunn P., Jewett P., Cuttitta F., Gazdar A., Minna J. (1986). Heterogeneity of cell surface antigen expression of human small cell lung cancer detected by monoclonal antibodies. Cancer Res..

[46] Shue Y.T., Lim J.S., Sage J. (2018). Tumor heterogeneity in small cell lung cancer defined and investigated in pre-clinical mouse models. Transl. Lung Cancer Res..

[47] Yang D., Denny S.K., Greenside P.G., Chaikovsky A.C., Brady J.J., Ouadah Y., Granja J.M., Jahchan N.S., Lim J.S., Kwok S. (2018). Intertumoral heterogeneity in SCLC is influenced by the cell type of origin. Cancer Discov..

[48] Arcaro A. (2015). Targeted therapies for small cell lung cancer: Where do we stand?. Crit. Rev. Oncol. Hematol..

[49] D’Amico D., Carbone D., Mitsudomi T., Nau M., Fedorko J., Russell E., Johnson B., Buchhagen D., Bodner S., Phelps R. (1992). High frequency of somatically acquired p53 mutations in small-cell lung cancer cell lines and tumors. Oncogene.

[50] Helin K., Holm K., Niebuhr A., Eiberg H., Tommerup N., Hougaard S., Poulsen H.S., Spang-Thomsen M., Norgaard P. (1997). Loss of the retinoblastoma protein-related p130 protein in small cell lung carcinoma. Proc. Natl. Acad. Sci. USA.

[51] George J., Lim J.S., Jang S.J., Cun Y., Ozretić L., Kong G., Leenders F., Lu X., Fernández-Cuesta L., Bosco G. (2015). Comprehensive genomic profiles of small cell lung cancer. Nature.

[52] Meder L., König K., Ozretić L., Schultheis A.M., Ueckeroth F., Ade C.P., Albus K., Boehm D., Rommerscheidt-Fuss U., Florin A. (2016). NOTCH, ASCL1, p53 and RB alterations define an alternative pathway driving neuroendocrine and small cell lung carcinomas. Int. J. Cancer.

[53] Rudin C.M., Durinck S., Stawiski E.W., Poirier J.T., Modrusan Z., Shames D.S., Bergbower E.A., Guan Y., Shin J., Guillory J. (2012). Comprehensive genomic analysis identifies SOX2 as a frequently amplified gene in small-cell lung cancer. Nat. Genet..

[54] Wistuba I.I., Gazdar A.F., Minna J.D. (2001). Molecular genetics of small cell lung carcinoma. Semin. Oncol..

[55] Byers L.A., Rudin C.M. (2015). Small cell lung cancer: Where do we go from here?. Cancer.

[56] Minna J.D., Roth J.A., Gazdar A.F. (2002). Focus on lung cancer. Cancer Cell.

[57] Rohr U.P., Rehfeld N., Pflugfelder L., Geddert H., Müller W., Steidl U., Fenk R., Gräf T., Schott M., Thiele K.P. (2004). Expression of the tyrosine kinase c-kit is an independent prognostic factor in patients with small cell lung cancer. Int. J. Cancer.

[58] Tamborini E., Bonadiman L., Negri T., Greco A., Staurengo S., Bidoli P., Pastorino U., Pierotti M.A., Pilotti S. (2004). Detection of overexpressed and phosphorylated wild-type kit receptor in surgical specimens of small cell lung cancer. Clin. Cancer Res..

[59] Baine M.K., Hsieh M.S., Lai W.V., Egger J.V., Jungbluth A., Daneshbod Y., Beras A., Spencer R., Lopardo J., Bodd F. (2020). Small cell lung carcinoma subtypes defined by ASCL1, NEUROD1, POU2F3 and YAP1: Comprehensive immunohistochemical and histopathologic characterization. J. Thorac. Oncol..

[60] Rudin C.M., Poirier J.T., Byers L.A., Dive C., Dowlati A., George J., Heymach J.V., Johnson J.E., Lehman J.M., MacPherson D. (2019). Molecular subtypes of small cell lung cancer: A synthesis of human and mouse model data. Nat. Rev. Cancer.

[61] McColl K., Wildey G., Sakre N., Lipka M.B., Behtaj M., Kresak A., Chen Y., Yang M., Velcheti V., Fu P. (2017). Reciprocal expression of INSM1 and YAP1 defines subgroups in small cell lung cancer. Oncotarget.

[62] Sonkin D., Thomas A., Teicher B.A. (2019). Are neuroendocrine negative small cell lung cancer and large cell neuroendocrine carcinoma with WT RB1 two faces of the same entity?. Lung Cancer Manag..

[63] Wagner P.L., Kitabayashi N., Chen Y.T., Saqi A. (2009). Combined small cell lung carcinomas: Genotypic and immunophenotypic analysis of the separate morphologic components. Am. J. Clin. Pathol..

[64] Gay C.M., Stewart C.A., Park E.M., Diao L., Groves S.M., Heeke S., Nabet B.Y., Fujimoto J., Solis L.M., Lu W. (2021). Patterns of transcription factor programs and immune pathway activation define four major subtypes of SCLC with distinct therapeutic vulnerabilities. Cancer Cell.

[65] Yu H.A., Arcila M.E., Rekhtman N., Sima C.S., Zakowski M.F., Pao W., Kris M.G., Miller V.A., Ladanyi M., Riely G.J. (2013). Analysis of tumor specimens at the time of acquired resistance to EGFR-TKI therapy in 155 patients with EGFR-mutant lung cancers. Clin. Cancer Res..

[66] Sequist L.V., Waltman B.A., Dias-Santagata D., Digumarthy S., Turke A.B., Fidias P., Bergethon K., Shaw A.T., Gettinger S., Cosper A.K. (2011). Genotypic and histological evolution of lung cancers acquiring resistance to EGFR inhibitors. Sci. Transl. Med..

[67] Marcoux N., Gettinger S.N., O’Kane G., Arbour K.C., Neal J.W., Husain H., Evans T.L., Brahmer J.R., Muzikansky A., Bonomi P.D. (2019). EGFR-mutant adenocarcinomas that transform to small-cell lung cancer and other neuroendocrine carcinomas: Clinical outcomes. J. Clin. Oncol..

[68] Niederst M.J., Sequist L.V., Poirier J.T., Mermel C.H., Lockerman E.L., Garcia A.R., Katayama R., Costa C., Ross K.N., Moran T. (2015). RB loss in resistant EGFR mutant lung adenocarcinomas that transform to small-cell lung cancer. Nat. Commun..

[69] Offin M., Chan J.M., Tenet M., Rizvi H.A., Shen R., Riely G.J., Rekhtman N., Daneshbod Y., Quintanal-Villalonga A., Penson A. (2019). Concurrent RB1 and TP53 alterations define a subset of EGFR-mutant lung cancers at risk for histologic transformation and inferior clinical outcomes. J. Thorac. Oncol..

[70] Iams W.T., Beckermann K.E., Almodovar K., Hernandez J., Vnencak-Jones C., Lim L.P., Raymond C.K., Horn L., Lovly C.M. (2019). Small cell lung cancer transformation as a mechanism of resistance to PD-1 therapy in KRAS-mutant lung adenocarcinoma: A report of two cases. J. Thorac. Oncol..

